# Acceptability of a Self-Guided Lifestyle Intervention Among Young Men: Mixed Methods Analysis of Pilot Findings

**DOI:** 10.2196/53841

**Published:** 2024-04-05

**Authors:** Jean Miki Reading, Melissa M Crane, Justin Guan, Ronston Jackman, Maria D Thomson, Jessica Gokee LaRose

**Affiliations:** 1 Department of Preventive Medicine Feinberg School of Medicine Northwestern University Chicago, IL United States; 2 Department of Family and Preventive Medicine Rush University Chicago, IL United States; 3 Department of Social and Behavioral Sciences School of Population Health Virginia Commonwealth University Richmond, VA United States

**Keywords:** digital health, gender, weight loss, health behaviors, low touch, obesity, obese, mixed methods analysis, lifestyle intervention, young men, men, effectiveness, digital tools, food intake, diet

## Abstract

**Background:**

Young men are vastly underrepresented in lifestyle interventions, suggesting a need to develop appealing yet effective interventions for this population.

**Objective:**

This study aimed to determine the acceptability of a self-guided lifestyle intervention designed specifically for young men (age: 18-35 years old).

**Methods:**

Semistructured interviews and surveys were completed by 14 men following completion of a remotely delivered, 12-week lifestyle intervention. The intervention included 1 virtual group session, digital tools, access to self-paced web- and mobile-based content, and 12 weekly health risk text messages. We quantitatively and qualitatively examined young men’s experiences with the intervention components of a remotely delivered, self-guided lifestyle intervention targeting weight loss. Data were integrated using convergent mixed methods analysis.

**Results:**

Men were a mean age of 29.9 (SD 4.9) years with a mean BMI of 31.0 (SD 4.5) kg/m^2^. The self-guided aspect was not acceptable, and a majority preferred more check-ins. Participants expressed a desire for a social aspect in future lifestyle interventions. All men found the focus on health risks appealing. A majority of men found the study-issued, Bluetooth-enabled scale acceptable.

**Conclusions:**

Acceptability of the self-guided lifestyle intervention was perceived as suboptimal by young men. The findings highlight the need to add intervention components that sustain motivation and provide additional social support for young men.

**Trial Registration:**

ClinicalTrials.gov NCT04267263; https://www.clinicaltrials.gov/study/NCT04267263

## Introduction

Young men with obesity during young adulthood have twice the mortality risk of men with a healthy BMI (kg/m^2^) [1]. Despite heightened risk, young men are underrepresented in lifestyle interventions targeting weight loss [2-5]. Low enrollment among young men may stem from their limited concern about weight gain [6,7] or the absence of lifestyle interventions designed to meet the specific needs of men [8]. Adapting interventions to align with the needs and preferences of young men [9,10] while also raising awareness about the risks associated with weight gain among this demographic [11] could potentially enhance engagement with weight loss.

Gender-specific lifestyle interventions indicate promise for engaging men to lose weight and include different features appealing to men (eg, sports-based or self-guided approach) [9,12-15]. Implementing a self-guided approach appears efficacious in producing initial weight loss and satisfaction among young men [14,16,17] and is consistent with young men’s preferences for convenient interventions [9]. When considering age, young adults demonstrate a preference for interventions that reduce intensity and promote autonomy [18]. In a pilot weight loss trial targeting young men, a self-guided approach, paired with health risk messaging, promoted modest weight loss compared with the control [11]. However, it remains unclear which specific elements are perceived as helpful in supporting weight loss for young men.

To improve young men’s engagement with weight loss, it is critical to adapt behavioral weight loss interventions based on their needs. Guidelines for behavioral intervention development are outlined in the Obesity-Related Behavioral Intervention Trials (ORBIT) model and recommend using an iterative process to reach optimal treatment outcomes for the target population [19]. The ORBIT model specifically notes using mixed methods approaches for defining and refining interventions and for feasibility pilot testing [19]. Prior to a rollout of a behavioral weight loss intervention, it is important to determine the acceptability of the intervention among users, which is often captured quantitatively through measures of satisfaction, attendance, and efficacy [20-23]. Satisfaction is a key construct to consider when developing an intervention, given that higher levels of satisfaction with an intervention are associated with favorable weight loss outcomes [24]. However, qualitative measures of satisfaction with interventions are limited in behavioral weight loss trials [24-26], especially as they relate to young men. As a result, our understanding of young men’s experiences with behavioral weight loss is limited.

Indeed, studies are needed that incorporate both quantitative and qualitative user feedback and experience with the intervention. To that end, we used a mixed methods approach to explore the experiences of young men who completed a 12-week lifestyle intervention designed specifically for this population. The primary aim of this paper was to explore young men’s satisfaction with specific intervention components of a self-guided lifestyle intervention to inform future development of interventions [11].

## Methods

### Study Design

This study was part of a randomized clinical trial in which 18 men received the intervention and 17 men were allocated to delayed treatment control. We report data for the 14 men who were randomized to the intervention and completed the 12-week follow-up visit, an exit interview, and a survey following the visit to provide feedback on the intervention components (retention rate: 14/18, 78%). The full design, protocol, and exclusion criteria are detailed elsewhere [11].

### Ethics Approval

All procedures were approved by the institutional review board at Virginia Commonwealth University (IRB# HM20015458).

### Recruitment

Men between the ages of 18 years and 35 years with a BMI of 25 kg/m^2^ to 45 kg/m^2^ were recruited across North America and locally in the greater Richmond, Virginia, area during a 2-month period (January 2021-March 2021) using unpaid recruitment advertisements distributed through email listservs, university postings, and researchmatch.org. Advertisements for the intervention emphasized that the lifestyle intervention was self-guided, included images of men exercising and a health risk message, and described some of the inclusion criteria (ie, BMI, age, men). Interested participants completed online screening via Research Electronic Data Capture (REDCap) to determine initial eligibility and were contacted by a member of the study team to schedule an orientation to learn more about the study and engage in an informed consent process. All participants provided written informed consent.

### Sample

Participants for the main trial [11] were eligible if they met the following inclusion criteria: (1) age 18 years to 35 years and (2) BMI between 25 kg/m^2^ and 35 kg/m^2^. Exclusion criteria included (1) medical contraindications to exercise without medical clearance, (2) a diagnosis of type 1 or 2 diabetes, (3) report of a heart condition, (4) a history of anorexia or bulimia nervosa, (5) report of compensatory behaviors in the last 3 months, (6) hospitalization for psychiatric conditions in last 12 months, (7) participation in another weight loss program (8) ≥5% weight loss in the last 3 months, (9) not able to read or speak English, (10) did not possess a mobile device, or (11) lived or resided outside of North America. Only participants assigned to the intervention arm were eligible for the current aims.

### Intervention

Men received the 12-week lifestyle intervention that was primarily self-guided and grounded in behavioral self-regulation [27] and health risk messaging guided by the extended parallel process model [28]. The intervention was remotely delivered and included 1 group session delivered via Zoom. The group session was followed by self-paced content accessible through a private intervention website, 12 weekly health risk text messages (automated and nonresponsive), and personalized feedback at baseline and 12 weeks based on assessment data. All participants were provided with a Bluetooth-capable scale. All intervention content included health risk messaging applying the extended parallel process model’s constructs (perceived susceptibility, perceived severity, self-efficacy, response efficacy) [28]. The messaging highlighted health risks specific to young men, including the association between obesity and cardiovascular disease, and evidence-based strategies for facilitating weight loss and mitigating cardiovascular disease risk. The baseline feedback report was delivered via email and included the participant’s current BMI, weight, 5% and 10% weight loss goals, daily calorie goal, and health risk messaging. The 12-week feedback report included the participant’s BMI, weight, percentage of weight loss achieved during the intervention, and health risk messaging. Each intervention component is described in greater detail in the following paragraphs.

The virtual group session occurred via Zoom at the start of the intervention. The session was 45 minutes and led by a licensed clinical psychologist with expertise in behavioral weight loss treatment. Men were provided with psychoeducation regarding health risks of obesity [29,30] and behavioral self-regulation principles [31]. Additionally, men received training in evidence-based behavior change techniques for managing weight [31] and engaged in small group experiential activities to apply and practice skills to increase self-efficacy. In addition, men received instructions on how to access the website and content covered during the session.

**Figure 1:**
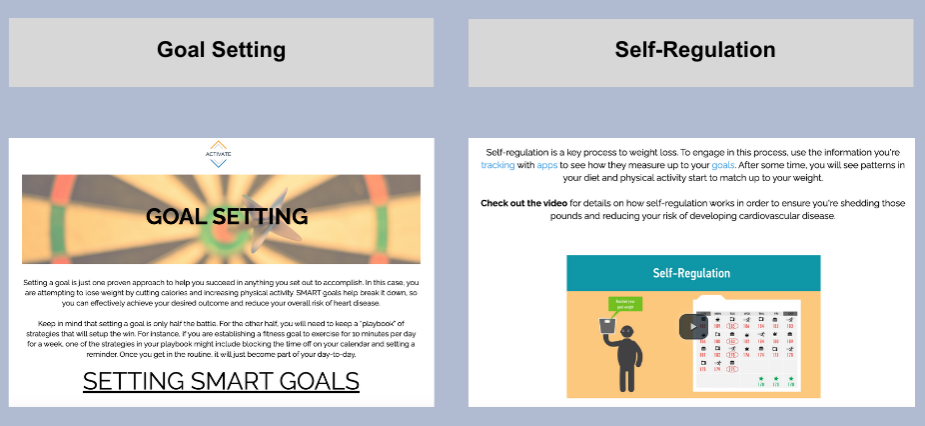
ACTIVATE Private Intervention Website and Features.

Digital tools included access to a private intervention website with evidence-based content, tools for self-monitoring, and a Bluetooth-capable scale. The intervention website was hosted through a private server and was accessible via web and mobile devices throughout the 12-week intervention. The website offered additional psychoeducation on healthy weight management, diet, and physical activity and behavioral change techniques for making changes to health behaviors. Content was adapted for health behaviors relevant to young men [9], which focused on improving fitness and reducing consumption of alcohol, sugar-sweetened beverages, processed meals and fast food, and foods high in fat. The website also housed links to publicly and commercially available online videos and apps for physical activity, self-monitoring diet, and meal preparation. See Figure 1 for example screenshots of the intervention website.

To reinforce extended parallel process model constructs, 12 weekly health risk text messages were sent throughout the intervention. For example, “Eating out and fast food can put men at high risk for heart disease due to high fat, calories, and sodium. Cutting back on fast food and the meals you eat away from home can lower your risk! Check out meal planning tips on [study website] for ways to reduce your risk.”

### Measures

At 12 weeks, participants were asked to report the acceptability of a variety of aspects of the intervention on a 7-point Likert scale. Participants rated aspects of the intervention (eg, male only, young adult only, self-guided) they found appealing upon joining the intervention (1=not appealing at all; 7=very appealing). Participants rated intervention components (group session, text messages, website) and certain features (length, frequency, relevance) perceived as helpful with weight loss (1=not helpful; 7=very helpful). Participants also rated intervention components and delivery methods not offered (eg, in-person meetings focused on physical activity, online meetings about diet).

A female PhD student (JMR, student investigator) with extensive interviewing experience conducted 14 interviews by phone in a private setting. On average, interviews lasted 15 (range: 8-29) minutes. The semistructured interview guide included open-ended questions about motivations for joining, satisfaction with the intervention, challenges and successes with the intervention, and recommendations for future interventions (Multimedia Appendix 1). Standardized probes were used to elicit responses about intervention components that were considered helpful or unhelpful for intervention goals. Interviews were audio-recorded and transcribed verbatim.

### Statistical Analysis

Descriptive statistics were computed for each question assessing intervention acceptability. Intervention components were deemed acceptable if the mean satisfaction score for each item was ≥5 (1 point above the central point of the 7-point scale) [32]. Descriptive statistics were computed using SPSS Version 27 (IBM Corp).

A directed content analysis was used to qualitatively code the semistructured interviews [33]. The coders consisted of 2 men with education in public health and behavioral medicine (MS and BS degrees) who were not investigators of the study. Both coders were trained by the student investigator with extensive experience in qualitative research (JMR). First, 4 transcripts were reviewed by the student investigator (JMR) to develop an initial codebook of primary and secondary codes. Transcripts were then analyzed in batches of 2 to 4 by the 2 coders, with minimal involvement by the student investigator. Intercoder reliability (kappa>0.80) and fidelity to the codebook were maintained during coding [34]. Incongruent codes were flagged, discussed, and reviewed at weekly meetings with the student investigator. All discrepancies were resolved through group consensus. Although data achieved saturation after 10 coded interviews [35], all data were coded. Coded categories were grouped by intervention components and aspects of participant satisfaction. Subcategories of each pre-identified theme were cross compared for word similarities to identify overlap in responses. Data were coded using NVivo 12.0 (QSR International).

JMR served as the student investigator and conducted this study as part of her doctoral dissertation in Social and Behavioral Sciences. Under guidance of the other senior authors, JMR made all final decisions throughout the study process. She considered her role as student investigator and her gender from conceptualization to the reporting of findings. Many factors, including time and financial constraints, were taken into account when determining who would collect and analyze the data. JMR collected the semistructured interview data due to availability, experience, and direct contact with participants throughout the study. After each interview, JMR documented her assumptions in memos. Given JMR’s vested interest in her doctoral dissertation, she took a minimal role in coding to mitigate potential bias in the qualitative findings. Her involvement in the qualitative data analysis primarily revolved around facilitating discussion on coding discrepancies and providing qualitative training.

A convergent mixed methods analysis [36] was used following standard guidelines [37]. Specific items from the quantitative data were integrated with corresponding themes related to intervention materials, in which qualitative data were embedded into quantitative data to explain acceptability ratings of intervention components.

## Results

Of the 18 men, 14 completed the 12-week follow-up visit after completing the intervention (retention: 78%). Participants’ mean age was 29.0 (SD 4.8) years, with a mean BMI of 31.0 (SD 4.7) kg/m^2^, and 29% (4/14) identified as racial and ethnic minorities. Demographic characteristics of the participants who completed the follow-up visit are displayed in Table 1. On average, participants lost –1.8% (SD 2.8%) of their initial body weight at 12 weeks (range: –9.5% to +1.3%). Results are described in 3 thematic areas in the following sections. Exemplary quotes from qualitative interviews corresponding to themes and subthemes are provided in Table 2.

**Table 1:** Sample characteristics (n=14).

Characteristics			Results, n (%)
**Age (years)**			
	18-25	3 (21)	
	26-35	11 (79)	
**Race^a^**			
	American Indian/White	1 (7)	
	Asian	1 (7)	
	White	11 (79)	
**Ethnicity**			
	Hispanic/Latino	4 (29)	
**Relationship status^a^**			
	Married	6 (43)	
	Single	6 (43)	
	Living with partner	1 (7)	
**Education**			
	Some college	2 (14)	
	College graduate	8 (57)	
	Postgraduate degree	4 (29)	

^a^Does not sum to 100% due to missing data not reported.

**Table 2:** Exemplary quotes by corresponding theme and subtheme.

Theme and subthemes			Exemplary quotes
**Appealing aspects of the intervention or motivations for joining**			
	Age and gender	“I think what specifically, uh, got me into it was the fact that it targeted young men [sic], my age group.”	
**Acceptability of intervention components**			
	Bluetooth scale and application	“The scale was really, really useful, actually, especially starting out because I could see, like, all the different metrics that I had no idea of before.”	
	Recommended applications	“I already use some of the nutrition tracking apps, but there was a workout app specifically, Fit On is the one that I kind of latched on to and I hadn’t heard of it before. And I use that as my primary source for exercise and kind of coming up with an actual exercise plan. And I use My Fitness Pal a little bit as well to check diet.”	
	Health risk text messages	“The text messages I found helpful [sic], as a reminder of the ultimate reason why I was doing this.” “I mean, uh, the uh, the text message that we got, it was good accountability, but it was also, that I can see how that could also just be something that you just kind of slough off because we just like, a here’s a fact.”	
**Preferences for an ideal intervention**			
	Social aspect	“Maybe a social component, more so than the accountability part, you know. Just like a shared experience kind of thing.”	
	Frequency of contact	“I would say like having, like, another check-in would maybe be good, like midway through or a couple of check-ins.”	

### Appealing Aspects of the Intervention or Motivations for Joining

During interviews, a desire to lose weight was the most common reason for joining the study (8/14, 57%). Other reasons for joining included meeting the advertised age and gender demographic (7/14, 50%), gaining knowledge (6/14, 43%), getting in shape (5/14, 36%), and the self-guided aspect (4/14, 29%).

I liked that it was targeted at my age group by my demographic in general. I feel like there are a lot of weight loss programs for other demographics. And this is the first one that I see that civically targeted, you know, and my age group.

Most participants did not report hesitation about joining the intervention. Those who reported a reluctance (3/14, 21%) about joining the intervention were mainly concerned about having enough time. The quantitative results indicate that the majority of participants found the intervention appealing and decided to join because of the emphasis on general lifestyle changes (13/14, 93%), focus on health risk (14/14, 100%), and weight loss (11/14, 79%). The lowest percentage of men rated the self-guided component (5/14, 36%) or minimal in-person contact (5/14, 36%) as reasons for joining. See Table 3.

**Table 3:** ACTIVATE intervention acceptability items.

Question			Overall results, mean (SD)		Percent rating ≥5.0^a^, n (%)
How satisfied were you with the overall ACTIVATE program that you received during the past 12 weeks?			4.2 (1.1)		5 (36)
How satisfied were you with what you achieved in the ACTIVATE program?			3.9 (.83)		4 (29)
Would you recommend the ACTIVATE program to other young men?			4.5 (1.6)		7 (50)
The information I learned in this program would be relevant to other men of my age who want to lose weight.			5.4 (1.2)		12 (86)
The length of the program was sufficient for a weight loss program targeting men my age (18-35).			4.4 (1.6)		7 (50)
**What parts of the program did you find appealing?**					
	Male only	5 (1.4)		7 (50)	
	Young adult only	5 (1.1)		8 (57)	
	Minimal in person	4.4 (1.7)		5 (36)	
	Self-guided	3.8 (1.8)		5 (36)	
	Focus on health risk	5.8 (.73)		14 (100)	
	Fitness	5.1 (1.1)		10 (71)	
	Diet	5.1 (1.1)		10 (71)	
	Weight loss	5.4 (1.0)		11 (79)	
	General lifestyle changes	5.9 (1.0)		13 (93)	
**How much did each of the following help you to lose weight?**					
	Group session	3.7 (1.9)		8 (57)	
	Intervention website	3.6 (1.6)		4 (29)	
	Meal plans	3 (1.2)		1 (7)	
	Text messages	4.3 (1.8)		7 (50)	
	Feedback	4.1 (1.0)		6 (43)	
	Scale	5.1 (1.4)		11 (79)	
	App to track food	4.0 (1.6)		6 (43)	
	App to track physical activity	4.5 (1.6)		8 (57)	
**Rating of website/group session**					
	The skills taught on the website helped me with my weight loss efforts.	4.1 (1.2)		5 (36)	
	The website content was motivating to me.	3.4 (1.3)		2 (14)	
	The information on the website was relevant to me.	4.1 (1.2)		7 (50)	
	The length of the group session was the right amount of time.	4.4 (1.2)		7 (50)	
	The strategies taught in the group session were helpful to me.	4.1 (1.3)		5 (36)	
	The information in the group session was motivating to me.	4.3 (1.1)		6 (43)	
	The information in the group session was relevant to me.	4.6 (1.2)		8 (57)	
**Rating of weekly text messages**					
	The messages were motivating to me.	5.0 (1.5)		9 (64)	
	The messages suggested strategies that were helpful to me.	4.2 (1.6)		9 (64)	
	The messages made me aware of the risks associated with weight gain.	5.4 (1.6)		11 (79)	
	The messages made me aware that I am at risk for cardiovascular disease.	4.9 (1.5)		8 (57)	
**Preferred additional features**					
	Add an online group component for discussion.	5.6 (1.4)		13 (93)	
	Add in-person group meetings focusing on diet.	4.9 (1.7)		9 (64)	
	Add online group meetings focusing on diet.	5.4 (1.4)		11 (79)	
	Add in-person group meetings focusing on physical activity.	4.6 (1.6)		9 (64)	
	Add online group meetings focusing on physical activity.	5.6 (1.3)		13 (93)	
	Add in-person group meetings focusing on muscle strengthening.	4.4 (1.6)		9 (64)	
	Add online group meetings focusing on muscle strengthening.	4.9 (1.4)		10 (71)	

^a^Percentage of participants rating intervention features acceptable (>5 on a 7-point scale).

### Acceptability of Intervention Components

Less than one-half (5/14, 36%) of the men rated the overall intervention acceptable (satisfaction >5). For specific components, the majority of the participants in the interviews mentioned the Bluetooth scales (11/14, 79%), recommended apps (9/14, 64%), and text messages (10/14, 71%) were helpful for weight loss.

For the digital tools, participants discussed finding the Bluetooth scale’s features, which included an app to track metrics and progress, helpful. One young man shared his thoughts on the Bluetooth scale:

The scale was really, really useful actually, especially starting out because I could see, like, all the different metrics that I had no idea of before.

Quantitative findings indicated the percentage of men who found the recommended apps as acceptable (rating ≥5) was lower than those in the qualitative findings. In particular, less than one-half of men (6/14, 43%) found the recommended applications to self-monitor diet acceptable. Over one-half of men (8/14, 57%) found the recommended applications to self-monitor physical activity acceptable. See Table 3.

A low percentage of men found the meal planning strategies (1/14, 7%) and the intervention website (4/14, 29%) as acceptable (rating ≥5). Less than one-half of the participants found the strategies taught in the group session helpful (5/14, 36%) and the website content motivating (2/14, 14%).

The quantitative data indicate over one-half of men found the text messages motivating (9/14, 64%), helpful (9/14, 64%), and raised awareness of the health risks of weight gain (11/14, 79%) and cardiovascular disease (8/14, 57%).

The qualitative data indicate participants found the weekly text messages served as good reminders. One-half (7/14, 50%) of the qualitative interviews indicate participants found the text messages of benefit. The other one-half (7/14, 50%) either did not find the messages helpful or had mixed feelings about them—some felt the content of the text messages did not have enough variety or were something that could be easily disregarded. One young man shared thoughts on the weekly health risk messages:

The text message that we got, it was good accountability, but it was also [sic] something that you just kind of slough off because it’s just like here’s a fact.

### Preferences for an Ideal Intervention

Of the participants, 71% (10/14) described a desire for a social aspect to the intervention, and almost all men (13/14, 93%) preferred an online group component for discussion. In particular, 93% (13/14) of men wanted an online group meeting focusing on physical activity. Participants discussed the desire for a message board or ongoing discussion with other participants to help with motivation or accountability throughout the intervention. Over one-third (5/14, 36%) also mentioned a desire for a more personalized experience and to receive more feedback (5/14, 36%). A participant shared:

Maybe a social component, more so than the accountability part, you know. Just like a shared experience kind of thing.

The majority (8/14, 57%) of participants wanted more contact from the intervention. For the most part, participants preferred to have midpoint or monthly online group check-ins, as opposed to weekly check-ins. 

## Discussion

### Principal Findings

This convergent mixed methods analysis provides insight into young men’s experiences with a self-guided lifestyle intervention to inform future intervention development for this vastly underrepresented population. We explored 3 key areas: motivations for joining and appealing aspects of the intervention, acceptability of specific intervention components, and preferences for an ideal intervention.

Motivations for joining and acceptable intervention components of a lifestyle intervention were key areas explored in this study. Qualitative and quantitative data surrounding motivations for joining and acceptable intervention components were fairly consistent. First, qualitative data indicated men’s interest in joining the study was related to age and gender. This underscores the importance of designing recruitment messaging specifically for young men as a way to enhance enrollment in lifestyle interventions [38]. Furthermore, the emphasis on health risks emerged as a key motivator for young men to participate in the lifestyle intervention. However, the existing literature on leveraging health risks to prompt health behavior change among men presents mixed findings. Although some findings suggest that fear of health complications [8] and a desire to improve health [16] act as motivators for engaging with weight loss behaviors, other research indicates that young men may prefer to avoid discussions about associated health risks [10]. It is plausible that the use of health risk messaging in recruitment advertisements resonated only with a subset of young men who chose to enroll in the study [39]. Further investigation is necessary to ascertain the generalizability of these findings and their implications on a broader scale.

The overall acceptability of the self-guided lifestyle intervention among young men was found to be suboptimal. However, young men reported high acceptability of the study-issued scale and companion app to track weight. Additionally, the online delivery method was well-received by men. Therefore, prioritizing remote delivery of lifestyle interventions for participants who are meeting weight loss goals could enhance accessibility, scalability, and convenience [40,41]. It is worth noting that young men perceived the self-guided aspect as somewhat “hands-off” and lacking personalization. Specifically, young men expressed dissatisfaction with the website, which did not offer new weekly content throughout the active intervention period. Collaborating with young men in future interventions to co-design weekly content might render higher appeal and engagement with the intervention.

Young men highlighted a preference for adding a social aspect in future interventions. Specifically, men reported wanting a shared similar experience—which is a reported benefit of using online platforms for sharing weight loss experiences [42]. Recent data underscore the potential for peer support to promote weight loss in a reduced intensity lifestyle intervention, but the majority of sample was adult women [43]. Of note, these preferences for a shared similar experience could be somewhat negated in standard behavioral weight loss interventions, which are predominantly women [2,44]. Qualitative data indicate men do not feel comfortable discussing men’s health issues and weight loss around women [45]. Thus, integrating a private online platform for young men to discuss relevant health issues might be one strategy for improving this population’s engagement with weight management. In addition to the desire for an online social platform, young men also reported a preference for online group sessions related to physical activity. This finding is consistent with formative data suggesting young men have a greater desire for interventions to focus on physical activity than young women, particularly as it relates to peer support and accountability [18]. Although physical activity is less effective at producing weight loss compared with diet alone [46], promoting physical activity upfront as a way to engage men could have a “spill-over” effect on other behaviors such as diet.

Last, incorporating social support via personalized weekly feedback derived from self-monitoring and goal progress is an evidence-based behavioral change technique [47] that might potentially boost motivation and engagement among young men. Despite men performing well in a self-guided lifestyle intervention targeting weight loss [14], young men in this study expressed a preference for monthly check-ins to aid in accountability. Therefore, future endeavors should consider testing different levels of intensity to determine the optimal level of support needed for men to achieve weight loss goals while also addressing time-related barriers faced by young men.

### Limitations

This study had several limitations. The sample was mostly non-Hispanic White with a college education. Given the racial and education disparities in both obesity prevalence and enrollment in behavioral weight loss interventions [48,49], more research is warranted to investigate the weight management needs and preferences of young men from marginalized racial and ethnic identities or men without a college education. These data were collected during COVID-19. Rapid shifts toward digital health interventions and the unique context of a global health pandemic may have impacted the findings in numerous ways (eg, desire for weight management, social connection). The treatment-seeking sample might not be generalizable to young men broadly. Moreover, we only interviewed men who returned for their follow-up visit. Thus, important elements for enhancing engagement were potentially missed in the present sample. Interviews were of shorter duration than a typical qualitative interview. However, given the specific goals and deductive design of the qualitative data and pairing of quantitative data, the depth of the participant responses was sufficient in addressing the paper objectives. Men who completed the interviews were slightly older than men who were lost to follow-up (30 years old vs 26 years old). Therefore, more work is needed to understand the preferences and experiences of emerging adult men specifically. Last, this study tested a “bundled” intervention package, which limited our ability to delineate effects of individual intervention components. Future research should apply rigorous factorial designs, such as the multiphase optimization strategy (MOST) [50], to delineate individual and combined effects and acceptability of intervention components to develop an optimized intervention package for young men.

### Strengths

This study had several strengths. To our knowledge, this is the first mixed methods study to report young men’s experiences with and the acceptability of a remotely delivered, self-guided lifestyle intervention targeting weight loss. In future work, fully embracing user-centered design will allow us to identify elements that improve the participant experience and related outcomes in lifestyle interventions [51]. Additionally, we had high agreement between coders. Last, we followed standard guidelines for the best practice of integrating the qualitative and quantitative findings [37]. Behavioral weight loss trials can benefit from a mixed methods design—using qualitative and quantitative data to complement inherent weaknesses in each, generate robust findings, and enhance validity [52].

### Conclusions

Our findings suggest that the acceptability of a self-guided approach was less than optimal among young men. To improve acceptability, potential enhancements might include incorporating online group sessions focused on physical activity, providing personalized feedback based on self-monitoring and goal setting, or implementing an online platform to foster peer support among young men. Further refinement of this lifestyle intervention is necessary before conducting a large-scale randomized controlled trial. A cost-efficient design, such as MOST [50], could be utilized to determine the most effective individual and combined intervention components offering the greatest clinical benefit while considering practical aspects such as cost and scalability. These interventions could enhance engagement among young men by augmenting the self-guided approach to include additional support such as optional online group exercise classes. These classes might be a relevant time to emphasize the importance of physical activity—as it relates to men’s health. Findings also suggest a social component could enhance accountability for men attempting weight loss. More research is needed to expand our understanding of young men’s experiences with weight management over a longer-term follow-up and engagement strategies for reaching emerging adults.

## Appendix

Multimedia Appendix 1 Semistructured interview guide.

## Abbreviations

MOST: multiphase optimization strategy
ORBIT: Obesity-Related Behavioral Intervention Trials
REDCap: Research Electronic Data Capture
