# Supplementary material for: Acceptability of a Self-Guided Lifestyle Intervention Among Young Men: Mixed Methods Analysis of Pilot Findings
Source: JMIR Form Res. 2024 Apr 5;8:e53841. doi: 10.2196/53841 (PMC11031701; doi:10.2196/53841)
Supplement: Multimedia Appendix 1 [file formative_v8i1e53841_app1.docx]

Supplementary Material 1. Semi-Structured Interview Guide

**Motivations to Join**

*Thinking back to when you first joined the study, what was appealing about the program that motivated you to join?*

[Probe for aspects of the study that were appealing to them]

[Male-only]

[Young adult only]

[Minimal in-person]

[Self-guided]

[Health risks—obesity, cardiovascular disease]

[Focus on fitness]

[Focus on diet]

[Focus on weight loss]

*Thinking back to when you first heard about the program, what questions did you have about the program?*

*[*Probe: *Please describe anything that made you hesitate or think twice about joining.]*

[Probe: *What could we say or do differently that would make the program more appealing to join?]*

*What do you think would be the most effective ways of advertising the program to other young men your age?*

**Intervention Content/Delivery**

*How satisfied were you with the program overall?*

[Probe for details about their experience with the program components]

[Probe for satisfaction with what they achieved in the program (i.e., their goals /

outcomes) and which things were most meaningful / important to them]

*Thinking about the intervention program, what parts of the program did you find the most helpful in meeting your goals? The least helpful? Anything else?*

[Probe for all program details—group session, toolkit (scale, self-monitoring, resources / apps, sample meal plans), handouts and videos on website, text messages, duration, time commitment, delivery mode]

*Which of the program recommendations did you struggle with while working towards your goals?*

[Probe for behaviors (self-monitoring diet, physical activity, or weight, meal planning, purchasing fruits and vegetables, reducing calorie intake, reducing alcohol, reducing how often I eat out)]

*What were some of the strategies used in your day-to-day living that helped you when trying to reach your goals?*

[Probe for behaviors (self-monitoring diet, physical activity, or weight, meal planning, purchasing fruits and vegetables, reducing calorie intake, reducing alcohol, reducing how often I eat out)]

*What were some of the suggested changes you found surprisingly challenging?*

*What aspects about the program would you suggest changing?*

*[Probe for suggestions that would be personally important]*

*If you were to talk to a friend interested in joining the program, what would you tell them about the program?*
